# Experimental malaria-associated acute respiratory distress syndrome is dependent on the parasite-host combination and coincides with normocyte invasion

**DOI:** 10.1186/s12936-018-2251-3

**Published:** 2018-03-05

**Authors:** Leen Vandermosten, Thao-Thy Pham, Hendrik Possemiers, Sofie Knoops, Evelien Van Herck, Julie Deckers, Blandine Franke-Fayard, Tracey J. Lamb, Chris J. Janse, Ghislain Opdenakker, Philippe E. Van den Steen

**Affiliations:** 1 Laboratory of Immunobiology, Department of Microbiology and Immunology, Rega Institute for Medical Research, KU Leuven-University of Leuven, Herestraat 49, box 1044, 3000 Leuven, Belgium; 2 Laboratory of Immunoregulation, VIB Center for Inflammation Research, Department of Internal Medicine, Ghent University, Technologiepark 927, 9052 Ghent, Belgium; 3 Leiden Malaria Research Group, Department of Parasitology, Leiden University Medical Center (LUMC), Albinusdreef 2, 2333 ZA Leiden, The Netherlands; 4 Department of Pathology, University of Utah, 15 N Medical Drive E, Salt Lake City, UT 84112 USA

**Keywords:** *Plasmodium berghei*, Malaria-associated acute respiratory distress syndrome, Lung, Reticulocyte, Normocyte

## Abstract

**Background:**

Malaria-associated acute respiratory distress syndrome (MA-ARDS) is a complication of malaria with a lethality rate of up to 80% despite anti-malarial treatment. It is characterized by a vast infiltration of leukocytes, microhaemorrhages and vasogenic oedema in the lungs. Previously, a mouse model for MA-ARDS was developed by infection of C57BL/6 mice with the Edinburgh line NK65-E of *Plasmodium berghei*.

**Results:**

Here, both host and parasite factors were demonstrated to play crucial roles in the development and severity of lung pathology. In particular, the genetic constitution of the host was an important determinant in the development of MA-ARDS. Both male and female C57BL/6, but not BALB/c, mice developed MA-ARDS when infected with *P. berghei* NK65-E. However, the New York line of *P. berghei* NK65 (NK65-NY) did not induce demonstrable MA-ARDS, despite its accumulation in the lungs and fat tissue to a similar or even higher extent as *P. berghei* NK65-E. These two commonly used lines of *P. berghei* differ in their red blood cell preference. *P. berghei* NK65-NY showed a stronger predilection for reticulocytes than *P. berghei* NK65-E and this appeared to be associated with a lower pathogenicity in the lungs. The pulmonary pathology in the C57BL/6/*P. berghei* NK65-E model was more pronounced than in the model with infection of DBA/2 mice with *P. berghei* strain ANKA. The transient lung pathology in DBA/2 mice infected with *P. berghei* ANKA coincided with the infection phase in which parasites mainly infected normocytes. This phase was followed by a less pathogenic phase in which *P. berghei* ANKA mainly infected reticulocytes.

**Conclusions:**

The propensity of mice to develop MA-ARDS during *P. berghei* infection depends on both host and parasite factors and appears to correlate with RBC preference. These data provide insights in induction of MA-ARDS and may guide the choice of different mouse-parasite combinations to study lung pathology.

## Background

Malaria is a world-wide disease with large areas of endemicity in sub-Saharan Africa, Asia and South America. Caused by infection with *Plasmodium* parasites, malaria affects around 200 million people, resulting in more than 400,000 deaths each year [1]. *Plasmodium* parasites are transmitted through the bites of infected female *Anopheles* mosquitos. The symptoms range from non-lethal uncomplicated malaria with fever, headache and vomiting, to life-threatening complications, such as severe malarial anaemia, cerebral malaria (CM), placental malaria and malaria-associated acute respiratory distress syndrome (MA-ARDS) [2]. Adults from high transmission areas are semi-immune and mostly protected against severe complications, including MA-ARDS. Thus, most cases of MA-ARDS are found in areas with low transmission of malaria and in non-immune travellers [3].

MA-ARDS has been found in patients infected with the two main species infecting humans, *Plasmodium falciparum* or *Plasmodium vivax*. MA-ARDS is also a main complication in *Plasmodium knowlesi*-infected patients [4], and some cases with *Plasmodium ovale* or *Plasmodium malariae* have been reported [5, 6]. The severity of MA-ARDS varies depending on the species of *Plasmodium* concerned, with the worst prognosis for *P. falciparum* infections [7, 8]. This may, in part, be related to distinct preferences for invading immature red blood cells (RBCs) or reticulocytes versus mature RBCs or normocytes [9, 10]. *P. vivax* and *P. ovale* merozoites only invade reticulocytes [11, 12]. This strongly reduces the number of cells available for invasion, since only between 1 and 2% of the total RBCs in the circulation are reticulocytes in healthy individuals. The reticulocyte restriction generally results in lower parasitaemias and has been linked to lower virulence compared to species which also invade normocytes, such as *P. falciparum*. However, lethal *P. vivax*-associated ARDS cases have been described in India [13].

MA-ARDS is characterized by an excessive pulmonary inflammation and breakdown of the alveolar-capillary membrane, resulting in overwhelming vasogenic oedema, alveolar flooding and severe hypoxaemia [3]. Adult patients with MA-ARDS have high mortality rates, up to 80%, despite anti-malarial treatment. The precise aetiology is poorly understood. *Post*-*mortem* histological analyses and data from experimental MA-ARDS models have indicated the occurrence of parasite sequestration and apoptosis of endothelial cells [14–19]. Sequestration of *P. falciparum*-infected RBCs is assumed to be highly important in the pathology of malaria [20]. Importantly, a massive inflammatory reaction occurs in the lungs during MA-ARDS [3, 15, 18], but the determinants which influence these phenomena remain poorly studied. This inflammation leads to accumulation of leukocytes in the pulmonary vasculature. Abundant macrophages and monocytes infiltrate the lungs of patients with MA-ARDS, in addition to lymphocytes and a few neutrophils [3]. This induced inflammation may lead to alveolar-capillary damage with a fatal outcome. Malaria pigment or haemozoin is abundantly present in infected RBCs (iRBCs), macrophages and monocytes in the lungs. The correlation of haemozoin deposition with pulmonary oedema and inflammatory cytokine induction in the lungs of mice with MA-ARDS suggests an important role for haemozoin in the pathogenesis [17].

To investigate the pathogenesis of MA-ARDS, several mouse models for MA-ARDS have been proposed in the past 10 years. Pulmonary oedema manifests in C57BL/6 mice infected with the *Plasmodium berghei* strain ANKA, the classical model for experimental cerebral malaria (ECM). This model has been used to investigate the pathogenesis of MA-ARDS [15, 21, 22]. However, the early and fulminant cerebral pathology in this model tends to limit the time-window available to study the pulmonary pathology. Therefore, several groups have developed alternative models of MA-ARDS. Epiphanio et al. [23] developed a model based on the infection of DBA/2 mice with *P. berghei* ANKA. These mice are entirely resistant to the cerebral pathology and approximately 50% of the mice develop malaria-associated acute lung injury (MA-ALI). Hee et al. [24] proposed the infection of C57BL/6 mice with *Plasmodium berghei* strain K173, which also causes lung pathology with increased pulmonary water content, although no protein-rich alveolar oedema could be documented. *P. berghei* K173 in C57BL/6 mice has also been used as a model of ECM with early death after infection due to cerebral pathology [25]. *Plasmodium chabaudi* AS-infected C57BL/6 mice develop very little lung oedema [18]. However, a recent study showed that *P. chabaudi* CB, a more virulent strain than *P. chabaudi* AS, does cause lung oedema associated with pulmonary inflammation and cell death [26].

Previously, a model for MA-ARDS was developed based on the infection of C57BL/6 mice with parasites of the NK65 strain of *P. berghei* [18]. This strain of *P. berghei* does not cause ECM in C57BL/6 mice, but leads to lethal pulmonary inflammation with protein-rich interstitial and alveolar oedema. The incidence of pulmonary pathology in this mouse model is high as more than 90% of infected mice develop MA-ARDS. However, infection of C57BL/6 mice with parasites of the NK65 strain has also been documented by other groups without any mention of pulmonary pathology and with sometimes highly different parasitaemia kinetics [27, 28]. Therefore, in this study it was investigated why these differences occur.

With mouse models of ECM, it is known that both parasite and host factors define the severity of disease. For example, parasites of the *P. berghei* ANKA strain induce ECM in C57BL/6 mice whereas cerebral complications are absent in BALB/c mice [29]. In rats, the age of the animal is an important factor in the development of cerebral complications with *P. berghei* ANKA infections [30]. Parasite factors also appear to play a role in ECM, since it has been demonstrated that various cloned lines derived from *P. berghei* ANKA induced differences in the pathology of ECM [29]. These differences in pathology emphasize the need for detailed data on the course of infections and disease in order to make a rational choice for certain parasite-host combinations. In contrast to ECM, less is known about lung disease in different mouse-parasite combinations.

In this study, the induction and severity of MA-ARDS in different mouse and parasite strains was analysed. This included comparisons of *P. berghei* NK65 infections in C57BL/6 versus BALB/c mice, as well as comparisons of infections with the two most commonly used lines of *P. berghei* NK65, the Edinburgh line (*P. berghei* NK65-E) and the New York line (*P. berghei* NK65-NY) in C57BL/6 mice. Furthermore, induction of MA-ARDS was compared between *P. berghei* NK65-infected C57BL/6 mice and *P. berghei* ANKA-infected DBA/2 mice. This study illustrates that, similar to ECM, both host and parasite genetics are important determinants of the induction and severity of MA-ARDS and that the lung pathology coincides with normocyte invasion. These data yield new insights into the pathogenesis and provide information for the choice of different mouse-parasite combinations to study MA-ARDS.

## Methods

### Ethical statement

All experiments were approved by the Animal Ethics Committee from the KU Leuven (License LA1210186 Belgium) and by the Animal Experiments Committee of the Leiden University Medical Center (DEC 12120). The Dutch Experiments on Animal Act is established under European guidelines (EU directive no. 86/609/EEC regarding the Protection of Animals used for Experimental and Other Scientific Purposes). All experiments were performed in accordance with relevant guidelines and regulations.

### Parasites and mice

Two different lines of the NK65 strain of *P. berghei* were used.

1. The ‘Edinburgh’ line of the NK65 strain (*P. berghei* NK65-E). This line is a kind gift of the late Prof. Dr. D. Walliker (University of Edinburgh, UK) [18]. The genome of a cloned line of this strain (1995cl2; RMgm-1115, http://www.pberghei.eu) has been sequenced [31]. This line has been used to generate the GFP-luciferase expressing line *P. berghei* NK65-E 2168cl2 as previously described [32] (Rmgm-4363; http://www.pberghei.eu).
2. The ‘New York’ line of the NK65 strain (*P. berghei* NK65-NY). This line was a kind gift of Prof. R. Ménard (Institut Pasteur, Unité de Biologie et Génétique du Paludisme, Paris, France). The genome of a cloned line of *P. berghei* NK65-NY has also been sequenced [31]. This line has been used to generate a cloned line expressing GFP-luciferase (1556cl1; RMgm-4364, http://www.pberghei.eu) that has been used in this study [31, 33].

In addition, the sequenced reference line of *P. berghei* ANKA (cl15cy1) was used [31].

Male and female C57BL/6 mice were obtained from Janvier (7–8 weeks old, Le Genest-Saint-Isle, France). For the bioluminescence imaging experiments performed at the LUMC, female C57BL/6 (6–7 weeks old) from Charles River were used. Mice were infected with *P. berghei* lines NK65-E, NK65-E 2168cl2, NK65-NY or *P. berghei* ANKA by intraperitoneal (IP) injection of 10^4^ infected RBCs [18, 31, 32, 34].

Mice were kept in a conventional animal house and drinking water was supplemented with 4-amino benzoic acid (0.375 mg/ml PABA, Sigma-Aldrich, Bornem, Belgium). For the bioluminescence experiments performed at LUMC, 4-amino benzoic acid was added to the mouse diet. Mice were sacrificed at indicated time points after infection by euthanasia with Dolethal (Vétoquinol, Aartselaar, Belgium; 200 mg/ml, IP injection of 50 µl).

### Determination of parasitaemia and reticulocytosis

A blood smear was taken at indicated time points and stained with Giemsa (1/10 dilution, VWR, Heverlee, Belgium) to obtain percentages of parasitaemia, reticulocytes and infected reticulocytes. Additionally, 1/500 diluted tail blood was counted using a Bürker chamber to obtain RBC concentrations, from which numbers of iRBCs, reticulocytes and infected reticulocytes per ml were calculated. Images of Giemsa-stained blood smears were taken at a magnification of 100 (100×/1.25 oil objective) with a Leica DM 2000 light microscope equipped with a DFC 295 camera (Leica Bond Max, Leica Microsystems, Diegem, Belgium).

For determination of parasitaemia by flow cytometry [35], tail blood (10 µl) from infected mice was collected in 1 ml of complete culture medium and cells were fixed in 1 ml of a 0.25% (v/v) glutaraldehyde solution in PBS at 4 °C and kept at 4 °C until analysis. These samples were stained with the DNA-specific fluorescent dye Hoechst-33258 (2 µmol/l) for 1 h at 37 °C and analysed with a a FACScan (LSR II, Becton–Dickinson). The fluorescence intensity and size (Forward Scatter; FSC; Sideward Scatter, SSC) of 50,000 cells per sample were measured and data analysis was performed using the FlowJo software (FlowJo, LLC, Ashland, USA).

### Quantification of lung pathology

Lung pathology was assessed by measuring the weights of unperfused small lungs and the protein and IgM concentrations in bronchoalveolar lavage fluid (BALF). To obtain BALF, the small lungs were pinched off, 500 μl of PBS was instilled in the large lungs through the trachea with a catheter and withdrawn after 30 s. This was repeated and both lavages were pooled. The BALF was centrifuged (10 min at 335 g, 4 °C), the protein concentration of the supernatant was determined by Bradford assay (Bio-Rad, Hercules, CA, USA) and IgM concentrations were determined by ELISA (Jackson Immunoresearch, Newmarket, UK). Cell pellets were resuspended and leukocytes and erythrocytes were counted in a Bürker chamber. The RBCs were lysed by NH_4_Cl treatment (0.83% NH_4_Cl, 10 mM Tris/HCl, pH 7.2) and the leukocytes were further mounted onto cytospin slides (Thermo Shandon, Cheshire, UK), which were prepared and stained with Hemacolor (Merck, Darmstadt, Germany). The percentages of lymphocytes, monocytes/macrophages, and neutrophils were determined by microscopy analysis of the cytospin slides.

### Determination of parasite accumulation in the lungs by luminescence imaging

Mice were IP injected with 10^4^ of the parasite lines *P. berghei* NK65-NY 1556cl1 and *P. berghei* NK65-E 2168cl2. Both lines express the fusion protein GFP-luciferase. In vivo imaging of luminescence was used to determine the relative parasite accumulation in the lungs [33]. Mice were anesthetized and received d-luciferin (100 mg/kg, Caliper Life Sciences) subcutanously in the scruff, 2 min before full body imaging. Luciferase activity was determined using an IVIS Lumina II in vivo Imaging System (Perkin Elmer Life Sciences, Waltham, USA). Quantitative analysis of bioluminescence of whole bodies was performed by measuring the luminescence signal intensity using the region of interest (lungs) settings of the Living Image 4.4 software.

### Determination of parasite accumulation in the lungs by qRT-PCR

After mechanical homogenization of the left lungs, total RNA was extracted (RNeasy mini kit, Qiagen, Hilden, Germany) and quantified (Nanodrop, Thermo Fischer, Aalst, Belgium). cDNA was synthesized (High capacity cDNA reverse transcription kit, Thermo Fischer), and quantitative reverse transcription-polymerase chain reaction (qRT-PCR) for mouse and parasite 18S was performed on 0.5 and 0.25 ng cDNA. The parasite 18S was determined with the following primer and probe sets, synthesized by Integrated DNA Technologies (IDT, Leuven, Belgium): TAACATGGCTTTGACGGGTAA (forward primer), TGCTGCCTTCCTTAGATGTG (reverse primer) and TCCGGAGAGGGAGCCTGAGAAATA (probe). *P. berghei* 18S data was normalized to the corresponding expression of the murine 18S RNA.

### Scoring of disease progression

As described previously [36], body weight, parasitaemia, and clinical parameters including spontaneous activity (SA), limb grasping (LG), body tone (BT), trunk curl (TC), pilo-erection (PE), shivering (Sh), abnormal breathing (AB), dehydration (D), incontinence (I) and paralysis (P) were evaluated daily to calculate a clinical score of disease severity. A disease score was given of 0 (absent) or 1 (present) for TC, PE, Sh and AB and 0 (normal), 1 (intermediate) or 2 (most serious) for the other parameters. The total clinical score was calculated by the following formula: SA + LG + BT + TC + PE + Sh + AB + 3 * (D + I + P). Mice were euthanized when humane endpoints were reached (clinical score of 15 or more) or at the indicated time points.

### Determination of RBC turnover

Mice were injected intravenously with 100 µl Sulfo-NHS-biotin (EZ-link sulfo-NHS-LC-biotin, Thermo Scientific, Rockford, US) in PBS at 10 mg/ml, which results in rapid and efficient biotinylation of RBCs. At several times thereafter (at least 4 h), tail blood was collected, RBC were counted in a haemocytometer and subsequently labelled with streptavidin-APC (eBiosciences, CA, USA) and analysed by flow cytometry (FACSCalibur, Beckton Dickinson, Erembodegem, Belgium). Non-biotinylated blood was used as a negative control.

### Statistical analysis

The Mann–Whitney U test was used to determine the statistical significance between two groups. Statistical analysis was done using the GraphPad Prism 7 software (GraphPad software, San Diego, USA). p-values smaller than 0.05 were considered statistically significant. p-values were defined as follows: *p < 0.05, **p < 0.01, ***p < 0.001, ****p < 0.0001. Occasional mice in which the parasite did not develop well (< 1% parasitaemia on day 9–10 post-infection (PI) for *P. berghei* NK65-E) were excluded. Unless otherwise specified, bars and curves represent the average with standard error of the mean. Asterisks without horizontal lines represent significant differences compared to the uninfected control group. Horizontal lines with asterisks on top indicate significant differences between groups.

## Results

### Brief history of used parasite lines and clones

*Plasmodium berghei* NK65 was originally isolated from an *Anopheles dureni millecampsi* mosquito in 1964 [37]. Subsequently, this parasite was sent to several laboratories over the world. Previously, it was shown that parasites from the ‘Edinburgh’ line of *P. berghei* NK65 cause MA-ARDS in C57BL/6 mice [17, 18, 32]. However, it was subsequently noticed that, parasites of the ‘New York’ line of *P. berghei* NK65 differed from *P. berghei* NK65-E in infection characteristics, such as development of parasitemia. The genomes of cloned lines of both *P. berghei* NK65-E and *P. berghei* NK65-NY have been sequenced [31]. Several transgenic clones of *P. berghei* NK65-E were generated, including a cloned line which expresses GFP-luciferase under the control of the schizont-specific *ama*-*1* promoter [32], which is designated as *P. berghei* NK65-E 2168cl2.

A comparable cloned line was generated using parasites of *P. berghei* NK65-NY. As in *P. berghei* NK65-E 2168cl2, a *gfp*-*luciferase* fusion gene under control of the *ama*-*1* promoter has been introduced into the silent *c/d type ssu*-*rRNA* locus of parasites of *P. berghei* NK65-NY [32]. This cloned line is designated as *P. berghei* NK65-NY 1556cl1.

Epiphanio et al. [23] described that DBA/2 mice infected with *P. berghei* ANKA results in MA-ALI in a proportion of the mice. Therefore, parasite growth and lung pathology levels were analysed and compared in C57BL/6 mice infected with *P. berghei* NK65-E or *P. berghei* NK65-NY and in DBA/2 mice infected with the sequence reference cloned line cl15cy1 of *P. berghei* ANKA [31].

### Comparison of lung pathology between C57BL/6 mice infected with *P. berghei* NK65-E or *P. berghei* NK65-NY and DBA/2 mice infected with *P. berghei* ANKA

**Fig. 1:**
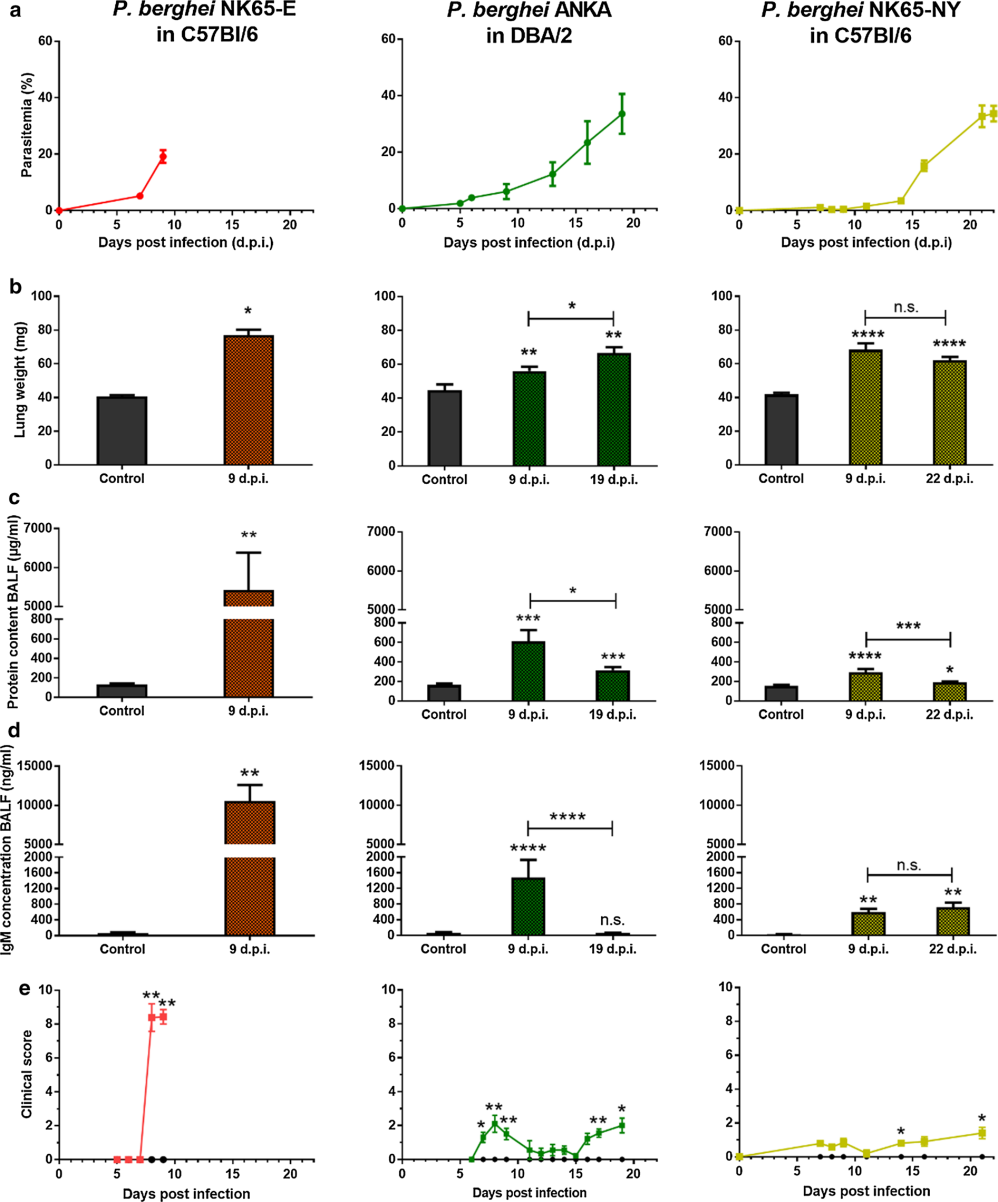
*P. berghei* NK65-E-infected C57BL/6 mice developed more severe lung pathology compared with *P. berghei* ANKA-infected DBA/2 mice and *P. berghei* NK65-NY-infected C57BL/6 mice. C57BL/6 mice were infected with *P. berghei* NK65-E or *P. berghei* NK65-NY and DBA/2 mice were infected with *P. berghei* ANKA. **a** Peripheral parasitaemia levels were determined through blood smears at the indicated time points (n = 7–32 per group). Mice were sacrificed at the indicated times and lung weight **b**, protein content **c** and IgM concentration **d** in BALF were measured (control: n = 4–10 per group, infected: n = 7–23 per group). **e** Clinical score was monitored throughout the course of infection (control: n = 4–8 per group, infected: 8–20 per group). Asterisks on top of the bars indicate significant differences compared to the uninfected control group. Horizontal lines with asterisks on top indicate significant differences between groups and horizontal lines with n.s. on top indicate no significant differences between groups

**Fig. 2:**
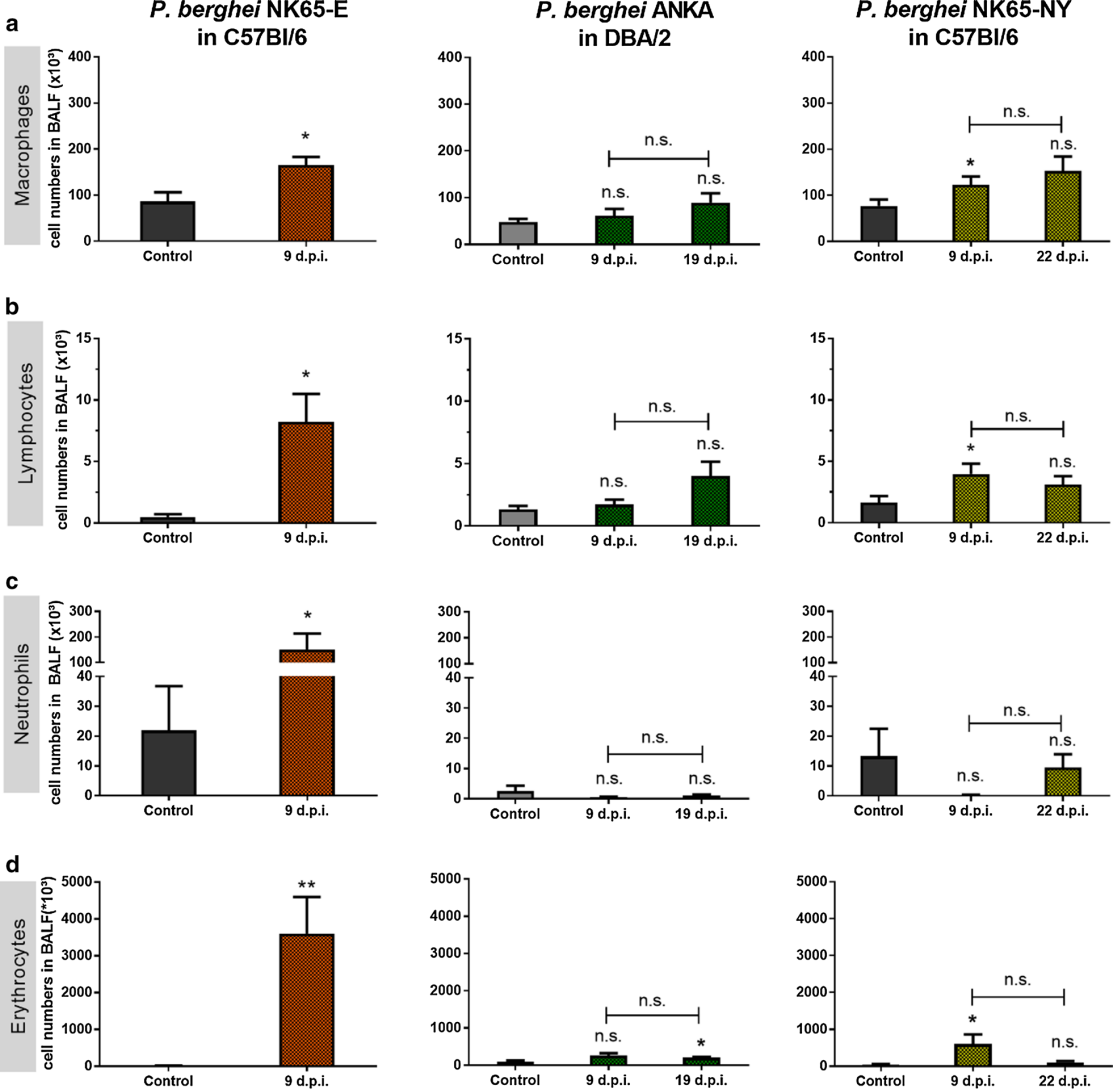
*P. berghei* NK65-E-infected C57BL/6 mice develop the highest alveolar leukocyte infiltration and haemorrhages. C57BL/6 mice were infected with *P. berghei* NK65-E or *P. berghei* NK65-NY and DBA/2 mice were infected with *P. berghei* ANKA. Mice were sacrificed at the indicated time points. **a** Macrophages, **b** lymphocytes and **c** neutrophils were differentially counted in cytospins of BALF (control: n = 4–10 per group, infected: n = 7–23 per group). **d** Erythrocytes were counted in BALF (control: n = 4–8 per group, infected: n = 6–18 per group). Asterisks on top of the bars indicate significant differences compared to the uninfected control group. Horizontal lines with n.s. on top indicate no significant differences between groups

The peripheral parasitaemia increased early in *P. berghei* NK65-E-infected C57BL/6 mice and led to morbidity and mortality around day 9 PI. *P. berghei* NK65-NY-infected C57BL/6 mice developed detectable, but low parasitaemia between 7 and 11 days PI and thereafter, parasitaemia increased rapidly before termination of the experiment. More severe lung pathology was observed in C57BL/6 mice infected with *P. berghei* NK65-E compared to *P. berghei* NK65-NY (Fig. 1). *P. berghei* NK65-E induced a full spectrum of pathology including an increase in lung weight, extensive alveolar oedema (as indicated by protein and IgM levels in the BALF), alveolar infiltration of immune cells and a high clinical score within 9 days PI, whilst mice infected with *P. berghei* NK65-NY developed a less pronounced lung pathology with minor increases of numbers of lymphocytes, macrophages and erythrocytes in the BALF at day 9 PI and subtle alveolar oedema. However, the changes in these parameters were considerably lower compared to mice infected with *P. berghei* NK65-E and did not result in a clearly increased clinical scores (Figs. 1, 2). Erythrocyte numbers in BALF of *P. berghei* NK65-E-infected C57BL/6 mice were greatly increased, indicating the occurrence of microhaemorrhages as previously described [18]. In *P. berghei* ANKA-infected DBA/2 mice, parasitaemia increased gradually from day 5 PI onwards until hyperparasitaemia at 3 weeks PI. An increase in lung weight and a transient and mild alveolar oedema was detected with no significant evidence of microhaemorrhages and a sparse infiltration of immune cells in the BALF (Figs. 1, 2). Only 10% of the *P. berghei* ANKA-infected DBA/2 mice died around 10 days PI, whereas in the other 90%, the alveolar oedema decreased after day 9 PI and mice recovered until they reached humane endpoints with hyperparasitaemia and anaemia after 19 days PI. A correspondingly transient and mild clinical score was noted around day 9 PI, which again increased at the end of the experiment.

Altogether, these results show that *P. berghei* NK65-E-infected C57BL/6 mice developed the most severe lung pathology, consistent with MA-ARDS. *P. berghei* ANKA-infected DBA/2 mice developed a less severe and transient form of lung pathology, and infection with *P. berghei* NK65-NY did not lead to overt lung pathology.

### The development of lung pathology is mouse strain-dependent

**Fig. 3:**
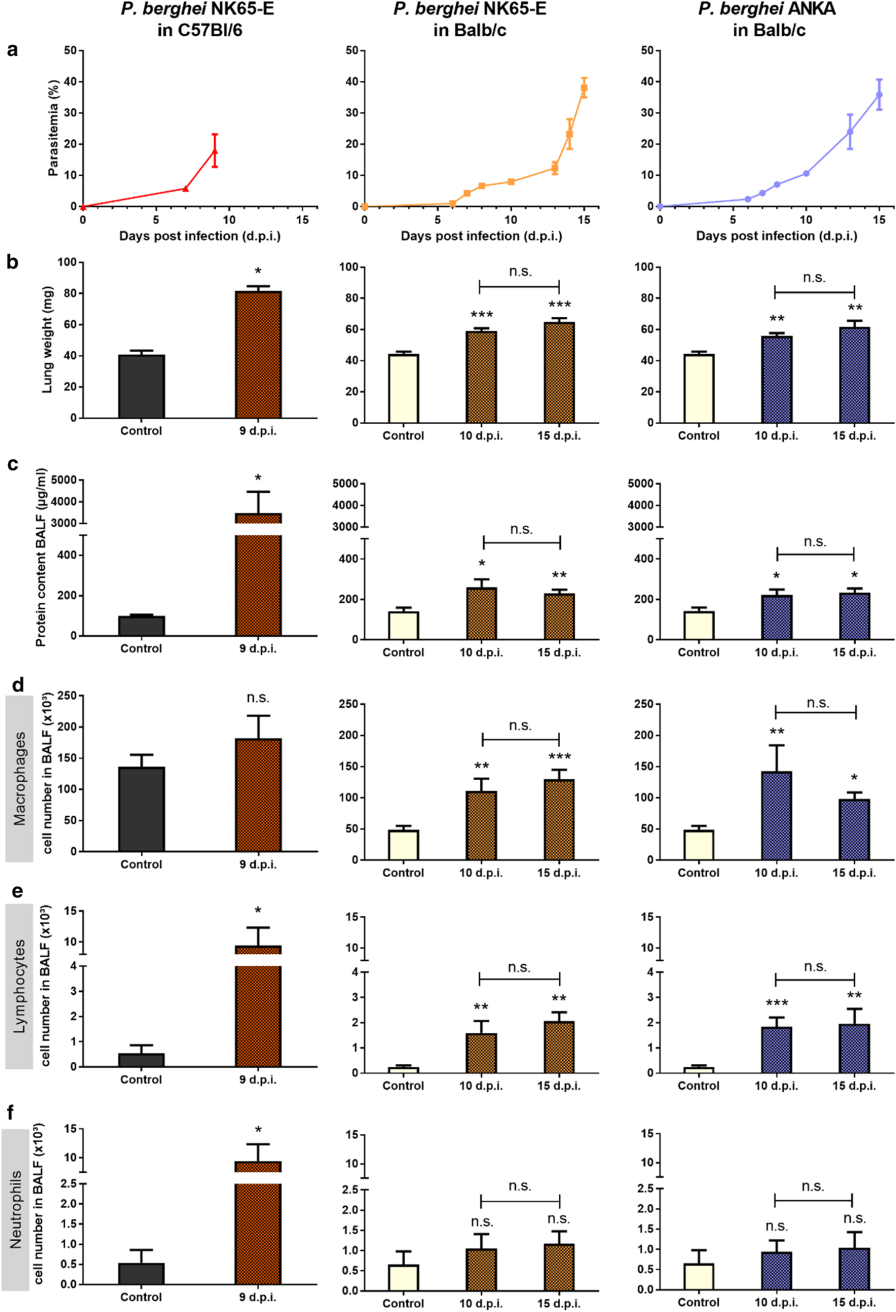
*P. berghei* NK65-E induces pronounced MA-ARDS in C57BL/6 mice but not in BALB/c mice. C57BL/6 mice were infected with *P. berghei* NK65-E, BALB/c mice were infected with *P. berghei* NK65-E or *P. berghei* ANKA. **a** Peripheral parasitaemia was determined through blood smears at the indicated time points (n = 3–21 per group). **b** Lung weight and **c** protein content in BALF were measured when mice were sacrificed at the indicated time points (control: n = 4–6 per group, infected: n = 5–12 per group). **d** Macrophages, **e** lymphocytes and **f** neutrophils were differentially counted in cytospins of BALF (control: n = 4–6 per group, infected: n = 5–12 per group). Asterisks on top of the bars indicate significant differences compared to the uninfected control group. Horizontal lines with n.s. on top indicate no significant differences between groups

To investigate the influence of the mouse strain on experimental MA-ARDS, BALB/c mice were infected with *P. berghei* NK65-E and compared to *P. berghei* NK65-E-infected C57BL/6 mice (Fig. 3). While *P. berghei* NK65-E-infected C57BL/6 mice developed MA-ARDS around day 9 PI at a parasitaemia above 10%, infected BALB/c mice developed a gradually increasing parasitaemia from day 6 PI onwards until hyperparasitaemia at 15 days PI. The lung weight increased significantly upon infection in both parasite-mouse combinations, but was most pronounced in *P. berghei* NK65-E-infected C57BL/6 mice. This was paralleled by an increase in alveolar oedema, which was only minor in BALB/c infected animals.

Infection of BALB/c mice with *P. berghei* NK65-E resulted in infiltration of macrophages and lymphocytes, but not neutrophils, into the lung (Fig. 3). However, the numbers of infiltrating macrophages and lymphocytes were lower compared to *P. berghei* NK65-E-infected C57BL/6 mice. Taken together, these data indicate that *P. berghei* NK65-E induces pronounced MA-ARDS, only in C57BL/6 mice and not in BALB/c mice. Additionally, *P. berghei* ANKA does not induce extensive lung pathology in BALB/c (Fig. 3), in contrast to its ability to induce lung oedema in DBA/2 mice (Fig. 1) or in C57BL/6 mice [15, 23].

### MA-ARDS pathogenesis is independent of sex in *P. berghei* NK65-infected C57BL/6 mice

**Fig. 4:**
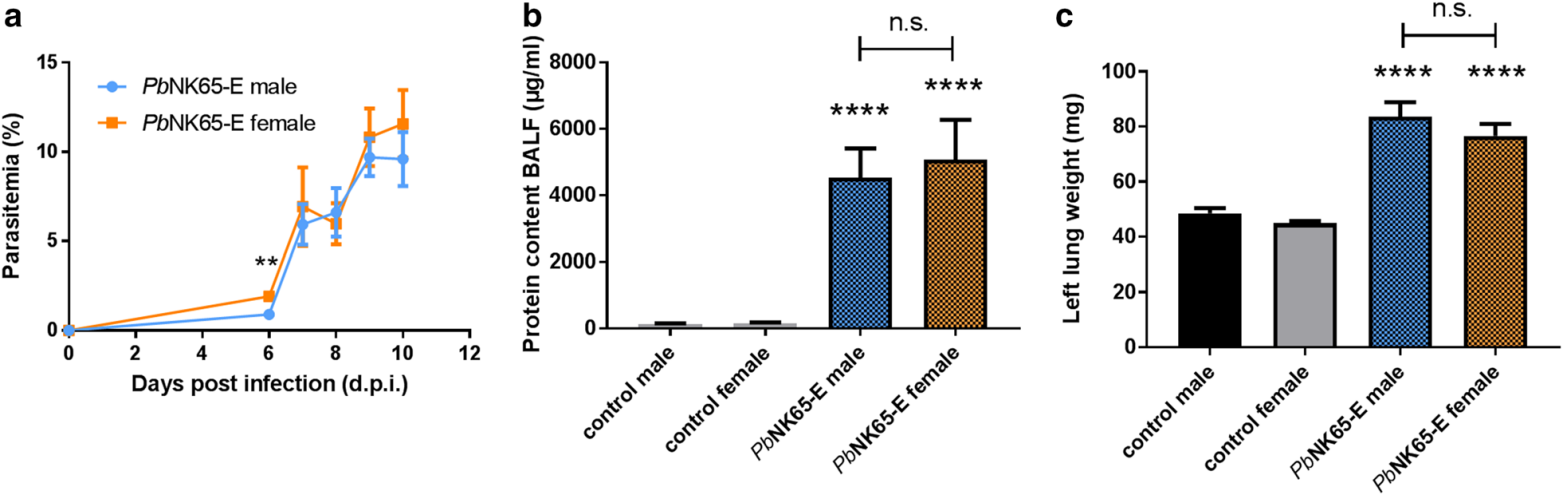
*P. berghei* NK65-E induces MA-ARDS in C57BL/6 mice independently of the sex. Male and female C57BL/6 mice were infected with *P. berghei* NK65-E (*Pb*NK65-E). **a** Peripheral parasitemia was determined through blood smears at indicated time points (n = 10–33 per group). **b** Upon dissection at 9–10 days PI, alveolar oedema was measured by protein determination (control: n = 15–17 per group, infected: n = 15–24 per group). **c** The lung weight was determined (control: n = 14–15 per group, infected: n = 18–30 per group). Asterisks on top of the bars indicate significant differences compared to the uninfected control group. Horizontal lines with n.s. on top indicate no significant differences between groups

Given previous studies that showed that male mice are less susceptible to some aspects of malaria pathogenesis than females [38–41], the course of *P. berghei* NK65-E infections and lung pathology between male and female C57BL/6 mice were compared (Fig. 4). Besides a minor difference on day 6 PI, the parasitaemia was not different between infected male and female C57BL/6 mice. Both male and female C57BL/6 mice developed lung pathology at 9–10 days PI in the *P. berghei* NK65-E model without significant differences (Fig. 4).

### The tissue tropism of *P. berghei* NK65-E and *P. berghei* NK65-NY are similar, but the kinetics of accumulation in the lungs differ

**Fig. 5:**
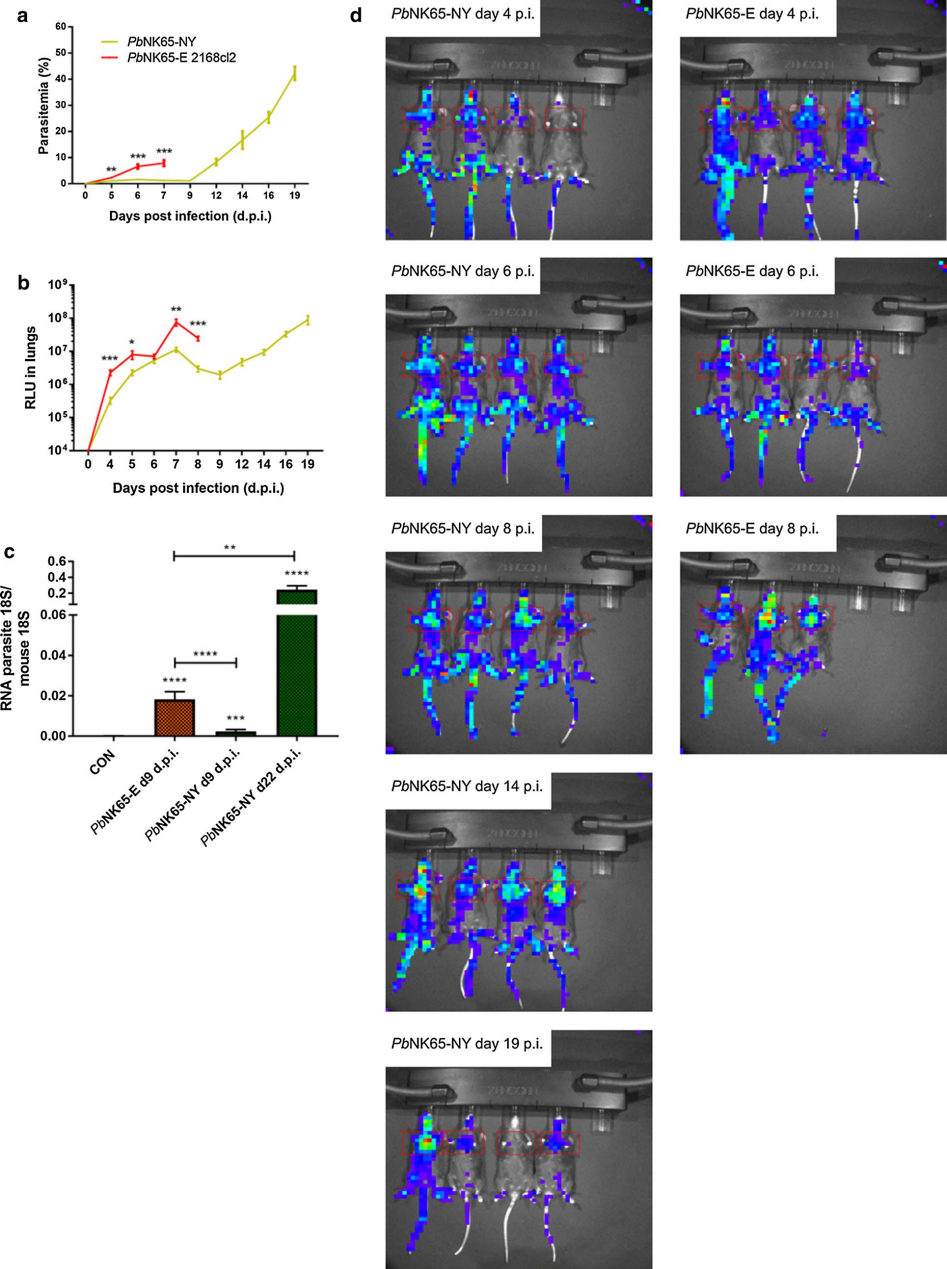
Tissue tropism of the *P. berghei* NK65-NY and *P. berghei* NK65-E parasite. C57BL/6 mice were infected with *P. berghei* NK65-NY (green, *Pb*NK65-NY) or *P. berghei* NK65-E 2168cl2 (red, *Pb*NK65-E). **a** Peripheral parasitemia was determined through flow cytometry at indicated time points. **b** The accumulation of *P. berghei* NK65 schizonts in lungs was measured and visualized through the bioluminescent features of the GFP-luc transfected parasites at the indicated time points and expressed as relative light units (RLU) (n = 8 per group). Asterisks indicate significant differences between groups. **c** C57BL/6 mice were infected with *P. berghei* NK65-NY or *P. berghei* NK65-E, and parasite loads in perfused large lungs were determined by quantifying parasite 18S RNA transcripts by RT-qPCR (n = 8–11 per group). Asterisks on top of the bars indicate significant differences compared to the uninfected control group. Horizontal lines with asterisks on top indicate significant differences between groups. **d** Whole-body bioluminescence images of representative infected mice are shown

Since peripheral parasitaemia in malaria excludes the iRBCs that are sequestering or accumulating in the organs, the parasite accumulation in the lungs of mice infected with *P. berghei* NK65-NY and *P. berghei* NK65-E was analysed. C57BL/6 mice were infected with GFP-luciferase expressing lines of these parasites (i.e. *P. berghei* NK65-NY and *P. berghei* NK65-E 2168cl2). These parasites express the fusion protein GFP-luciferase under control of a schizont-specific promotor, which allows visualization and quantification of schizonts through its luciferase activity by in vivo whole body imaging [31, 32]. A gradual increase in schizont accumulation until day 8 PI was seen in the lungs and umbilical fat tissue of *P. berghei* NK65-E 2168cl2 infected mice which develop MA-ARDS on day 7–8 PI (Fig. 5) [32]. In *P. berghei* NK65-NY-infected mice, which did not develop MA-ARDS, schizont accumulation increased gradually in the lungs and umbilical fat tissue at a reduced rate till day 7 PI, followed by a small drop and again a gradual increase until day 19 PI. Furthermore, parasites were quantified by qRT-PCR of parasite 18S in perfused lungs of *P. berghei* NK65-E and *P. berghei* NK65-NY-infected mice, as an accurate marker for parasite accumulation (Fig. 5c). On day 9 PI, parasite accumulation in the perfused lungs of *P. berghei* NK65-NY infected mice was significantly lower compared to *P. berghei* NK65-E infected mice, which confirmed the bioluminescence data. Moreover, the accumulation of *P. berghei* NK65-NY at day 22 PI was higher than with *P. berghei* NK65-E at day 9 PI, in accordance to the higher parasitemia. These data suggested that the distribution of *P. berghei* NK65-E and *P. berghei* NK65-NY parasites over different organs in the body are similar, and hence suggest that these parasite lines do not show major differences in their tissue tropism. However, the kinetics of the parasite accumulation is parasite-dependent and parallels the peripheral parasitaemia. Based on the luminescence data, the level of schizont accumulation in the lungs in *P. berghei* NK65-NY infected mice on day 19 PI was similar to that in *P. berghei* NK65-E 2168cl2 infected mice on day 7–8 PI (Fig. 5). However, this did not result in MA-ARDS in the *P. berghei* NK65-NY-infected mice, in contrast with the pronounced lung pathology in the *P. berghei* NK65-E-infected mice (Fig. 1). This suggests that pulmonary parasite accumulation alone is not enough to explain the pathology and that additional factors, possibly related to the capacity of infecting normocytes, contribute to the pathogenesis.

### Dynamics of reticulocytosis and RBC turnover in *P. berghei* NK65-E- and *P. berghei* NK65-NY-infected C57BL/6 mice and in *P. berghei* ANKA-infected DBA/2 mice

**Fig. 6:**
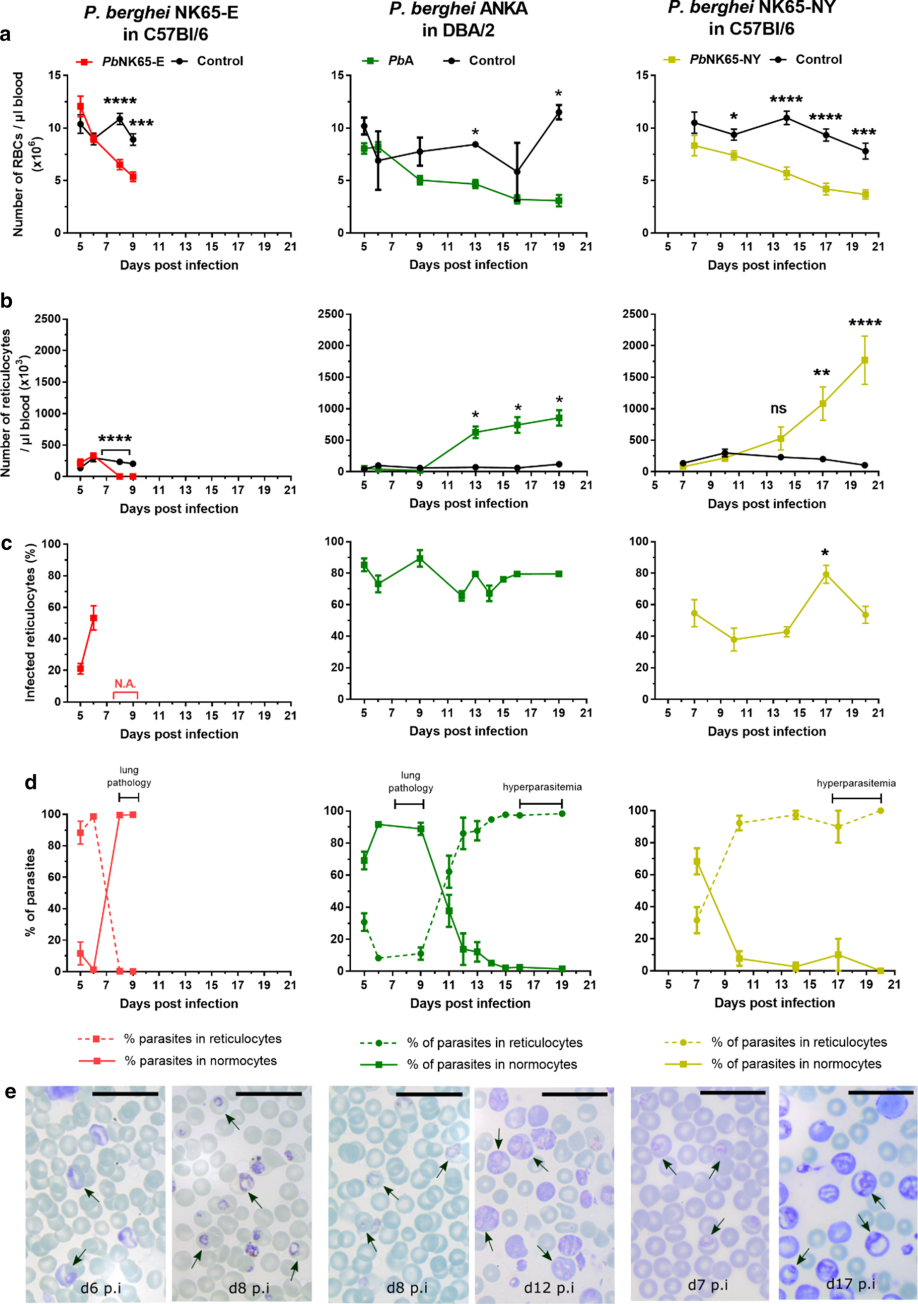
*P. berghei* NK65-E shifts from reticulocytes to normocytes and *P. berghei* ANKA and *P. berghei* NK65-NY from normocytes to reticulocytes. C57BL/6 mice were infected with *P. berghei* NK65-E (*Pb*NK65-E) or *P. berghei* NK65-NY (*Pb*NK65-NY), and DBA/2 mice were infected with *P. berghei* ANKA (*Pb*A). **a** Number of RBCs and **b** reticulocytes per µl blood, **c** percentages of infected reticulocytes and **d** percentages of parasites in reticulocytes and normocytes were determined at the indicated time points (control: n = 4 per group, infected: n = 8–12 per group). **e** Pictures from the Giemsa-stained smears are shown to illustrate the preference of the parasites for normocytes or reticulocytes. Arrows indicate infected RBCs and the black line represents 20 µm. Asterisks indicate significant differences between groups

Upon infection of C57BL/6 mice, *P. berghei* NK65-E and *P. berghei* NK65-NY displayed a different ability to invade RBCs of different ages. Initially, *P. berghei* NK65-E appeared to prefer invading reticulocytes. After the first week of infection, all available reticulocytes had been depleted and *P. berghei* NK65-E grew exponentially from 7 days PI onwards (Fig. 1). This coincided with a switch whereby parasites of the *P. berghei* NK65-E strain were found to invade mainly normocytes (Fig. 6). These observations indicate that *P. berghei* NK65-E has a predilection for invading reticulocytes, but that it can also infect normocytes in the absence of sufficient reticulocytes. This switch from reticulocyte to normocyte invasion coincided with the increase of the clinical score (Fig. 1). Mild anaemia developed shortly before *P. berghei* NK65-E-infected C57BL/6 mice died from MA-ARDS (Fig. 6).

Similar to *P. berghei* NK65 infections, *P. berghei* ANKA infection in DBA/2 mice demonstrated a relative preference for reticulocytes. Parasitaemia rose above 0.1% at 5 days PI and continued to increase steadily until around 30% at 19 days PI (Fig. 1). Reticulocyte numbers were relatively low in the control mice and in infected mice during the first phase of infection (up to day 9 PI) (Fig. 6). During this period, around 80% of the reticulocytes were infected. However, since parasite numbers were higher than reticulocyte numbers, between 75 and 100% of the parasites resided in normocytes (Fig. 6). RBC numbers decreased during the course of infection with a 62% reduction on day 19 compared to day 5 PI (Fig. 6). After 9 days PI, reticulocyte numbers increased, presumably as a consequence of the erythropoietic response to anaemia, and the parasite concomitantly infected more reticulocytes than normocytes (Fig. 6). This reflects the preference of *P. berghei* ANKA for reticulocytes, although it can infect normocytes when only low numbers of reticulocytes are available. Interestingly, the switch from normocyte to reticulocyte corresponded to a striking decrease in clinical score (Fig. 1), suggesting that the parasite is less pathogenic when it infects reticulocytes.

In *P. berghei* NK65-NY-infected C57BL/6 mice, parasitaemia remained below 3% until 14 days PI (Fig. 1). At 7 days PI, parasites were detected in both reticulocytes and normocytes. Later during the infection, the number of reticulocytes started to increase and from 14 day PI onwards, high reticulocyte numbers were observed. This was concomitant with a high parasitaemia, and parasites were detected almost exclusively in reticulocytes (Fig. 6). Anaemia also continued to aggravate with a 50% reduction of RBC numbers on day 20 compared to day 7 PI (Fig. 6). Of the reticulocytes, between 40 and 80% was infected throughout the course of infection (Fig. 6). These data suggest that NK65-NY has a strong predilection for reticulocytes and that it cannot develop increased parasitaemia in normocytes, in contrast to *P. berghei* NK65-E and *P. berghei* ANKA.

**Fig. 7:**
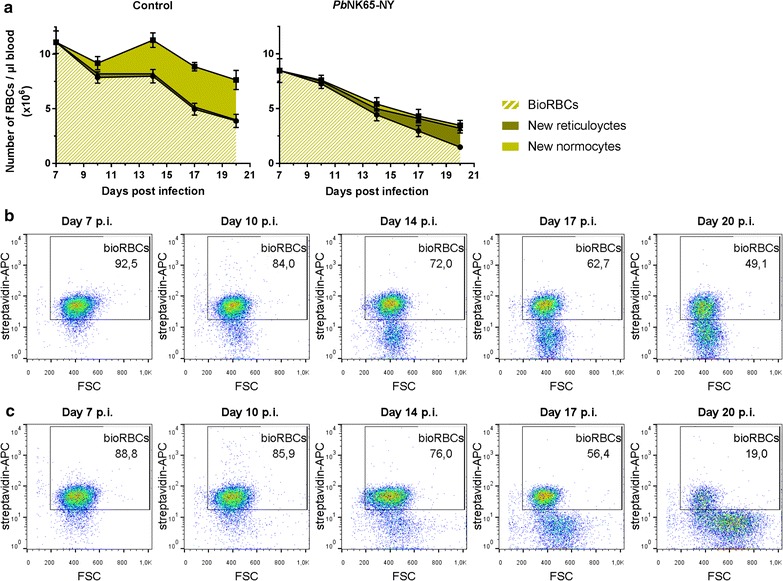
RBC turnover indicates predilection of *P. berghei* NK65-NY parasites for reticulocytes in C57BL/6 mice. C57BL/6 mice were infected with *P. berghei* NK65-NY (*Pb*NK65-NY). RBCs were in vivo biotin-labeled 7 days PI and the percentage of biotinylated (BioRBCs) versus non-biotinylated cells was quantified by flow cytometry (control: n = 7 per group, infected: n = 8 per group). **a** Number of RBCs per µl of blood. The non-biotinylated RBCs were subdivided into reticulocytes and normocytes, according to microscopy countings of the Giemsa-stained blood smears. **b** Representative FACS plots of RBCs of control mice (upper row) and infected mice (lower row), showing the percentage of BioRBCs and the forward scatter (FSC)

To further demonstrate the reticulocyte-predilection of *P. berghei* NK65-NY during the complete course of infection in C57BL/6 mice, the RBC dynamics that result in anaemia were investigated. The RBC turnover was determined by in vivo biotin-labelling of RBCs 7 days PI. By quantifying the percentage of biotinylated versus non-biotinylated cells using flow cytometry, it is possible to visualize both the clearance of RBCs from the circulation and the generation of new RBCs from the bone marrow (Fig. 7). In the period between day 7 PI and the end of the experiment (day 20 PI), the labelled RBCs (bioRBCs) disappeared at a similar rate in uninfected controls and infected mice, despite the relatively high parasitaemia after day 11 PI. This demonstrated that the turnover of the bioRBCs, which corresponded to the RBCs already present at day 7 PI and consisting of mainly normocytes, did not increase during infection. This suggests that few or no labelled normocytes were destroyed by *P. berghei* NK65-NY (Fig. 7) and supports the idea that this parasite has a pronounced predilection for reticulocytes over normocytes.

In contrast with the comparable turnover of bioRBCs, the number of unlabelled RBCs, which correspond to RBCs newly formed after the biotinylation on day 7 PI, was lower in the infected mice compared to the uninfected controls (Fig. 7). Calculation of the number of reticulocytes based on the microscopical analysis of the blood smears revealed that in uninfected mice, only a small proportion of the newly formed RBCs were reticulocytes, which is in agreement with the rapid maturation of reticulocytes into normocytes (typically less than 1 day). However, in the infected mice, the unlabelled RBCs consisted mainly of reticulocytes instead of normocytes. This suggests that the reticulocytes in the infected mice are immediately infected and destroyed before they can mature into (unlabelled) normocytes (Fig. 7). This explanation is plausible given that one erythrocytic cycle of the parasite, from invasion to rupture lasts 24 h and given the high percentage of infected reticulocytes observed. These data also indicate that, in this model, destruction of reticulocytes by the parasite contributes to the lack of replenishment of new normocytes, which is a main cause of anaemia. Interestingly, the newly formed non-biotinylated RBCs from infected mice are larger in size from day 14 PI onwards, compared to reticulocytes from uninfected control mice, as detected both by light microscopy analysis and by analysis of forward scatter by flow cytometry. This suggests that reticulocytes are released in a more immature stage from the bone marrow or spleen in the infected mice from day 14 PI onwards [42], in line with a strong stimulation of erythropoiesis by the anaemia.

## Discussion

The development of MA-ARDS in mice appears highly dependent on both mouse and parasite strains. In the current study, it is shown that MA-ARDS develops with *P. berghei* NK65-E, whereas *P. berghei* NK65-NY causes only minor pulmonary oedema. Also, the genetic background of the host is crucial, as MA-ARDS develops in C57BL/6 mice but not in BALB/c mice. Furthermore, DBA/2 mice infected with *P. berghei* ANKA also develop lung pathology, but clearly to a lower degree as indicated by the lower alveolar oedema. The latter parasite-mouse combination results in lethality in only a fraction of the DBA/2 mice, whereas the other mice survive the transient phase of the lung pathology and develop hyperparasitaemia and anaemia later on.

The differential susceptibility of mouse strains to specific malaria complications is a common observation. It is well-known that *P. berghei* ANKA-induced ECM develops quickly in C57BL/6 mice and CBA/J mice, whereas BALB/c and DBA/2 mice are resistant to this condition [29, 43]. Infection of BALB/c with *Plasmodium yoelii* XL is lethal, but the same parasite in DBA/2 mice is not lethal, causing a transient parasitaemia [44]. Furthermore, infection of C57BL/6 or BALB/c mice with *P. chabaudi* AS results in a transient parasitaemia wave together with limited liver pathology; however, *P. chabaudi* AS causes lethal anaemia in A/J mice [45–47]. Several genetic factors play a role in the susceptibility of the host to the parasite. In particular, a genetic background biased to more severe Th1 immune responses may be more prone to the development of Th1-mediated pathology. This is illustrated by the increased CD8^+^ T cell activation and CXCR3 expression in C57BL/6 mice versu*s* BALB/c mice, resulting in ECM in the C57BL/6 mice only [43]. However, the timing and potency of the Th1 response can have differential effects depending on the infecting parasite. Whilst DBA/2 mice are resistant to the development of *P. berghei* ANKA-induced cerebral pathology, a more potent Th1 response mounted to *P. yoelii* results in a more efficient parasite control in DBA/2 mice compared to BALB/c mice [44].

Differences between parasite strains and laboratory lines are crucial in determining the severity of malarial disease. Previous reports have highlighted differences in virulence between clones of *P. berghei* ANKA for the induction of ECM [29]. Also, several cloned lines of *P. chabaudi* have been shown to induce different levels of anaemia and weight loss across several different murine backgrounds [48, 49]. Of relevance to this study, *P. chabaudi* CB is more virulent than *P. chabaudi* AS and induces pulmonary pathology [26]. Importantly, multiple passaging of parasites may affect their virulence, as was shown for *P. chabaudi* AS [50]. Prominent differences especially occur between parasites that were recently passaged through mosquitos and parasites that were passaged several times by blood transfer. The latter procedure enhances the virulence, and this appears to be related to a more restricted pattern of expression of the *pir* multigene family [51].

Here, it is shown that two laboratory lines of *P. berghei* NK65 differed strikingly in terms of MA-ARDS induction. These lines presumably originate from one strain since it was isolated from a single infected mosquito [37]. Most likely, the passaging of parasites of this strain in different laboratories has resulted in the pronounced phenotypic differences between *P. berghei* NK65-E and *P. berghei* NK65-NY. The genetic diversity between these two lines is limited, as determined by genome sequencing recently [31]. However, these data suggested a more pronounced reticulocyte predilection of *P. berghei* NK65-NY compared to *P. berghei* NK65-E. Some *P. berghei* NK65-NY parasites, including ring stages, were observed in normocytes at 7 days PI, suggesting that *P. berghei* NK65-NY has the ability to invade normocytes. However, the replication in normocytes appeared inefficient as parasitaemias above 3% were only observed when sufficient numbers of reticulocytes were available. Furthermore, in vivo analysis of the RBC turn-over confirmed that only very few or no normocytes were destroyed by *P. berghei* NK65-NY. This reticulocyte predilection was associated with the absence of lung pathology. The switch from infection of reticulocytes to normocytes is important for rapid and early increases in *P. berghei* NK65-E parasitaemia, which coincides with the occurrence of pulmonary pathology.

*P. berghei* is known to have preference for invading reticulocytes versus normocytes [52]. Interestingly, Hopp et al. [53] described recently that berghepain-1 is a crucial protease for the infectivity of normocytes, as berghepain-1 knockout of *P. berghei* ANKA was completely reticulocyte-restricted. The growth of this parasite was limited by the availability of reticulocytes, similar to the here described findings with *P. berghei* NK65-NY, and could even be enhanced by treating the mice with phenylhydrazine, which results in RBC lysis and secondary reticulocytosis. Similarly, chloroquine-resistant *P. berghei* ANKA is also reticulocyte-restricted and produces less haemozoin, explaining the decreased sensitivity to chloroquine [54]. Plasmepsin-4/berghepain-2 double knockout parasites produce almost no haemozoin, are chloroquine-insensitive and are reticulocyte-restricted. They display a severe delayed growth phenotype and diminished ECM pathogenicity [55]. Furthermore, induction of reticulocytosis by administration of erythropoietin is also protective in the *P. berghei* ANKA model of cerebral malaria, although direct anti-inflammatory and neuroprotective actions of erythropoietin may be involved as well [56–58]. Lastly, the importance of being a generalist in RBC invasion can be highlighted by *P. yoelii*, as the non-lethality in the reticulocyte-invading XNL strain is contrasted with the lethal generalist XL strain [59]. Here, it was found that MA-ARDS occurs in *P. berghei* NK65-E infected C57BL/6 mice, when the parasite switches from invading mainly reticulocytes to invading mainly normocytes, and that the lung pathology in *P. berghei* ANKA-infected DBA/2 mice resolves when the parasite switches from invading normocytes to invading reticulocytes. The reasons why infected reticulocytes appear less pathogenic than normocyte invasion are currently unknown and may include a lower content of pathogenic factors in infected reticulocytes compared to infected normocytes, or the induction of a disease-limiting response by infected reticulocytes but not by infected normocytes.

Altogether, these data suggest that distinct lines of the *P. berghei* NK65 strain exist with completely different characteristics regarding the MA-ARDS pathology, and that the *P. berghei* parasite is more pathogenic when it resides in normocytes compared to reticulocytes, even at similar parasitemia levels, iRBC load and distribution in different organs. This association between reticulocyte invasion and absence of lung pathology is in line with human infections, as MA-ARDS is relatively less lethal upon infection with the reticulocyte-restricted *P. vivax* than with *P. falciparum* [9]. Future investigations are needed to clarify the mechanisms that underly the differences in MA-ARDS induced by different parasite lines. The data in this study emphasize the importance of choosing the correct mouse-parasite combinations to study mechanisms underlying MA-ARDS.

## Abbreviations

AB: abnormal breathing
BALF: bronchoalveolar lavage fluid
BT: body tone
CM: cerebral malaria
D: dehydration
ECM: experimental cerebral malaria
I: incontinence
IP: intraperitoneal
iRBC: infected red blood cell
LG: limb grasping
MA-ALI: malaria-associated acute lung injury
MA-ARDS: malaria-associated respiratory distress syndrome
P: paralysis
PE: pilo-erection
PI: post-infection
RBC: red blood cell
SA: spontaneous activity
Sh: shivering
TC: trunk curl

## References

[1] WHO. World malaria report 2016. Geneva: World Health Organization; 2016. http://www.who.int/malaria/publications/world-malaria-report-2016/report/en/.

[2] Trampuz A, Jereb M, Muzlovic I, Prabhu RM (2003). Clinical review: severe malaria. Crit Care.

[3] Van den Steen PE, Deroost K, Deckers J, Van Herck E, Struyf S, Opdenakker G (2013). Pathogenesis of malaria-associated acute respiratory distress syndrome. Trends Parasitol.

[4] Daneshvar C, Davis T, Cox-Singh J, Rafa’ee MZ, Zakaria SK, Divis PC (2009). Clinical and laboratory features of human *Plasmodium knowlesi* infection. Clin Infect.

[5] Haydoura S, Mazboudi O, Charafeddine K, Bouakl I, Baban TA, Taher AT (2011). Transfusion-related *Plasmodium ovale* malaria complicated by acute respiratory distress syndrome (ARDS) in a non-endemic country. Parasitol Int.

[6] Senpinar-Brunner N, Eckert T, Wyss K (2009). Near-fatal multiple organ dysfunction syndrome induced by *Plasmodium malariae*. Emerg Infect Dis.

[7] Sarkar S, Saha K, Das CS (2010). Three cases of ARDS: an emerging complication of *Plasmodium vivax* malaria. Lung India.

[8] Manyindo N, Simo D, Arora S, Unegbu U, Oyiriaru D, Wills R (2011). A case of successfully treated acute respiratory distress syndrome complicating *Plasmodium falciparum* malaria. J Natl Med Assoc.

[9] Mohan A, Sharma SK, Bollineni S (2008). Acute lung injury and acute respiratory distress syndrome in malaria. J Vector Borne Dis.

[10] Taylor WRJ, Hanson J, Turner GDH, White NJ, Dondorp AM (2012). Respiratory manifestations of malaria. Chest.

[11] McKenzie FE, Jeffery GM, Collins WE (2002). *Plasmodium vivax* blood-stage dynamics. J Parasitol.

[12] Kerlin DH, Gatton ML (2013). Preferential invasion by *Plasmodium* merozoites and the self-regulation of parasite burden. PLoS ONE.

[13] Kochar DK, Das A, Kochar SK, Saxena V, Sirohi P, Garg S (2009). Severe *Plasmodium vivax* malaria: a report on serial cases from Bikaner in northwestern India. Am J Trop Med Hyg.

[14] Valecha N, Pinto RGW, Turner GDH, Kumar A, Rodrigues S, Dubhashi NG (2009). Histopathology of fatal respiratory distress caused by *Plasmodium vivax* malaria. Am J Trop Med Hyg.

[15] Lovegrove FE, Gharib SA, Peña-Castillo L, Patel SN, Ruzinski JT, Hughes TR (2008). Parasite burden and CD36-mediated sequestration are determinants of acute lung injury in an experimental malaria model. PLoS Pathog.

[16] Liu M, Amodu AS, Pitts S, Patrickson J, Hibbert JM, Battle M (2012). Heme mediated STAT3 activation in severe malaria. PLoS ONE.

[17] Deroost K, Tyberghein A, Lays N, Noppen S, Schwarzer E, Vanstreels E (2013). Hemozoin induces lung inflammation and correlates with malaria-associated acute respiratory distress syndrome. Am J Respir Cell Mol Biol.

[18] Van Den Steen PE, Geurts N, Deroost K, Van Aelst I, Verhenne S, Heremans H (2010). Immunopathology and dexamethasone therapy in a new model for malaria-associated acute respiratory distress syndrome. Am J Respir Crit Care Med.

[19] Punsawad C, Viriyavejakul P, Setthapramote C, Palipoch S (2015). Enhanced expression of Fas and FasL modulates apoptosis in the lungs of severe *P. falciparum* malaria patients with pulmonary edema. Int J Clin Exp Pathol.

[20] MacPherson GG, Warrell MJ, White NJ, Looareesuwan S, Warrell DA (1985). Human cerebral malaria. A quantitative ultrastructural analysis of parasitized erythrocyte sequestration. Am J Pathol.

[21] Anidi IU, Servinsky LE, Rentsendorj O, Stephens RS, Scott AL, Pearse DB (2013). CD36 and fyn kinase mediate malaria-induced lung endothelial barrier dysfunction in mice infected with *Plasmodium berghei*. PLoS ONE.

[22] Lagasse HAD, Anidi IU, Craig JM, Limjunyawong N, Poupore AK, Mitzner W (2016). Recruited monocytes modulate malaria-induced lung injury through CD36-mediated clearance of sequestered infected erythrocytes. J Leukoc Biol.

[23] Epiphanio S, Campos MG, Pamplona A, Carapau D, Pena AC, Ataíde R (2010). VEGF promotes malaria-associated acute lung injury in mice. PLoS Pathog.

[24] Hee L, Dinudom A, Mitchell AJ, Grau GE, Cook DI, Hunt NH (2011). Reduced activity of the epithelial sodium channel in malaria-induced pulmonary oedema in mice. Int J Parasitol.

[25] Curfs J, Van der Meide P, Billiau A, Meuwissen J, Eling W (1993). *Plasmodium berghei*: recombinant interferon-gamma and the development of parasitemia and cerebral lesions in malaria-infected mice. Exp Parasitol.

[26] Lin J, Sodenkamp J, Cunningham D, Deroost K, Tshitenge TC, McLaughlin S (2017). Signatures of malaria-associated pathology revealed by high-resolution whole-blood transcriptomics in a rodent model of malaria. Sci Rep.

[27] Oliveira-Lima OC, Bernardes D, Pinto MCX, Arantes RME, Carvalho-Tavares J (2013). Mice lacking inducible nitric oxide synthase develop exacerbated hepatic inflammatory responses induced by *Plasmodium berghei* NK65 infection. Microbes Infect.

[28] Lacerda-Queiroz N, Lima OCO, Carneiro CM, Vilela MC, Teixeira AL, Carvalho AT (2011). *Plasmodium berghei* NK65 induces cerebral leukocyte recruitment in vivo: an intravital microscopic study. Acta Trop.

[29] Amani V, Boubou MI, Pied S, Marussig M, Walliker D, Mazier D (1998). Cloned lines of *Plasmodium berghei* ANKA differ in their abilities to induce experimental cerebral malaria. Infect Immun.

[30] Adam E, Pierrot C, Lafitte S, Godin C, Saoudi A, Capron M (2003). The age-related resistance of rats to *Plasmodium berghei* infection is associated with differential cellular and humoral immune responses. Int J Parasitol.

[31] Otto TD, Böhme U, Jackson AP, Hunt M, Franke-Fayard B, Hoeijmakers WAM (2014). A comprehensive evaluation of rodent malaria parasite genomes and gene expression. BMC Biol.

[32] Pham T-T, Verheijen M, Vandermosten L, Deroost K, Knoops S, Van den Eynde K (2017). Pathogenic CD8+ T cells cause increased levels of VEGF-A in experimental malaria-associated acute respiratory distress syndrome, but therapeutic VEGFR inhibition is not effective. Front Cell Infect Microbiol.

[33] Braks J, Aime E, Spaccapelo R, Klop O, Janse CJ, Franke-Fayard B (2012). Bioluminescence imaging of *P. berghei* schizont sequestration in rodents. Methods Mol Biol.

[34] Franke-Fayard B, Janse CJ, Cunha-Rodrigues M, Ramesar J, Büscher P, Que I (2005). Murine malaria parasite sequestration: CD36 is the major receptor, but cerebral pathology is unlinked to sequestration. Proc Natl Acad Sci USA.

[35] Lelliott PM, Lampkin S, McMorran BJ, Foote SJ, Burgio G (2014). A flow cytometric assay to quantify invasion of red blood cells by rodent *Plasmodium parasites* in vivo. Malar J.

[36] Vandermosten L, De Geest C, Knoops S, Thijs G, Chapman KE, De Bosscher K (2017). 11Β-Hydroxysteroid dehydrogenase type 1 has no effect on survival during experimental malaria but affects parasitemia in a parasite strain-specific manner. Sci Rep.

[37] Yoeli M, Most H (1965). Studies on sporozoite-induced infections of rodent malaria. I. The pre-erythrocytic tissue stage of *Plasmodium berghei*. Am J Trop Med Hyg.

[38] Li C, Corraliza I, Langhorne J, Corraliza S (1999). A defect in interleukin-10 leads to enhanced malarial disease in *Plasmodium chabaudi chabaudi* infection in mice. Infect Immun.

[39] Cernetich A, Garver LS, Jedlicka AE, Klein PW, Kumar N, Scott AL (2006). Involvement of gonadal steroids and gamma interferon in sex differences in response to blood-stage malaria infection. Infect Immun.

[40] Nagayasu E, Nagakura K, Akaki M, Tamiya G, Makino S, Nakano Y (2002). Association of a determinant on mouse chromosome 18 with experimental severe *Plasmodium berghei* malaria. Infect Immun.

[41] Kamis A, Ibrahim J (1989). Effects of testosterone on blood leukocytes in *Plasmodium berghei*-infected mice. Parasitology Res.

[42] Malleret B, Xu F, Mohandas N, Suwanarusk R, Chu C, Leite JA (2013). Significant biochemical, biophysical and metabolic diversity in circulating human cord blood reticulocytes. PLoS ONE.

[43] Van den Steen PE, Deroost K, Van Aelst I, Geurts N, Martens E, Struyf S (2008). CXCR3 determines strain susceptibility to murine cerebral malaria by mediating T lymphocyte migration toward IFN-gamma-induced chemokines. Eur J Immunol.

[44] Wang QH, Liu YJ, Liu J, Chen G, Zheng W, Wang JC (2009). *Plasmodium yoelii*: assessment of production and role of nitric oxide during the early stages of infection in susceptible and resistant mice. Exp Parasitol.

[45] Brugat T, Cunningham D, Sodenkamp J, Coomes S, Wilson M, Spence PJ (2014). Sequestration and histopathology in *Plasmodium chabaudi* malaria are influenced by the immune response in an organ-specific manner. Cell Microbiol.

[46] Chang K-H, Tam M, Stevenson MM (2004). Modulation of the course and outcome of blood-stage malaria by erythropoietin-induced reticulocytosis. J Infect Dis.

[47] Barthelemy M, Vuong PN, Gabrion C, Petit G (2004). *Plasmodium chabaudi chabaudi* chronic malaria and pathologies of the urogenital tract in male and female BALB/c mice. Parasitology.

[48] Taylor LH, Mackinnon MJ, Read AF, Apr N, Mackinnon J, Read F (1998). Virulence of mixed-clone and single-clone infections of the rodent malaria *Plasmodium chabaudi*. Evolution.

[49] Mackinnon MJ, Read AF (1999). Genetic relationships between parasite virulence and transmission in the rodent malaria *Plasmodium chabaudi*. Evolution.

[50] Mackinnon MJ, Gaffney DJ, Read AF (2002). Virulence in rodent malaria: host genotype by parasite genotype interactions. Infect Genet Evol.

[51] Spence PJ, Jarra W, Lévy P, Reid AJ, Chappell L, Brugat T (2013). Vector transmission regulates immune control of *Plasmodium* virulence. Nature.

[52] Deharo E, Coquelin F, Chabaud AG, Landau I (1996). The erythrocytic schizogony of two synchronized strains of *Plasmodium berghei*, NK65 and ANKA, in normocytes and reticulocytes. Parasitol Res.

[53] Hopp CS, Bennett BL, Mishra S, Lehmann C, Hanson KK, Lin J-W (2017). Deletion of the rodent malaria ortholog for falcipain-1 highlights differences between hepatic and blood stage merozoites. PLoS Pathog.

[54] Pisciotta JM, Scholl PF, Shuman JL, Shualev V, Sullivan DJ (2017). Quantitative characterization of hemozoin in *Plasmodium berghei* and *vivax*. Int J Parasitol Drugs Drug Resist.

[55] Lin J, Spaccapelo R, Schwarzer E, Sajid M, Annoura T, Deroost K (2015). Replication of *Plasmodium* in reticulocytes can occur without hemozoin formation, resulting in chloroquine resistance. J Exp Med.

[56] Kaiser K, Texier A, Ferrandiz J, Buguet A, Meiller A, Latour C (2006). Recombinant human erythropoietin prevents the death of mice during cerebral malaria. J Infect Dis.

[57] Wiese L, Hempel C, Penkowa M, Kirkby N, Kurtzhals JAL (2008). Recombinant human erythropoietin increases survival and reduces neuronal apoptosis in a murine model of cerebral malaria. Malar J.

[58] Wei X, Li Y, Sun X, Zhu X, Feng Y, Liu J (2014). Erythropoietin protects against murine cerebral malaria through actions on host cellular immunity. Infect Immun.

[59] Schaecher K, Kumar S, Yadava A, Vahey M, Ockenhouse CF (2005). Genome-wide expression profiling in malaria infection reveals transcriptional changes associated with lethal and nonlethal outcomes. Infect Immun.

